# Intraoperative functional brain mapping for glioma surgery: a comprehensive review of the University of California San Francisco mapping protocol

**DOI:** 10.1007/s11060-026-05664-7

**Published:** 2026-06-13

**Authors:** Jia-Shu Chen, Brandon Bergsneider, Alexander F. Haddad, Ramin A. Morshed, Shawn L. Hervey-Jumper, Jacob S. Young, Mitchel S. Berger

**Affiliations:** Department of Neurological Surgery, University of California San Francisco, San Francisco, CA USA

**Keywords:** Awake craniotomy, Brain tumor, Functional brain mapping, Language mapping, Motor mapping

## Abstract

**Purpose:**

Intraoperative functional brain mapping is an essential and intricate technique in modern-day glioma surgery. This article is not a review of the literature but of the technical protocol at our institution that has evolved over the recent decades to the current time and is intended to highlight details that enable us to perform maximal safe resection of gliomas.

**Methods:**

Prior to surgery, anatomical and functional imaging protocols are obtained to determine the tumor to be resected within its anatomical and functional environment. Preoperative assessments are used to determine which mapping procedures and tasks are most appropriate. Cortical and subcortical motor and language mapping using low and high frequency stimulation paradigms are applied when appropriate during resection. Methods to interpret findings and troubleshoot issues are reviewed herein.

**Results:**

All preoperative imaging including magnetic resonance imaging, magnetoencephalography of functional cortex, and diffusion tensor imaging of subcortical tracts are uploaded into the neuronavigation station and used throughout surgery for guidance. The decision to continue with tumor resection is based on constant feedback from the mapping paradigms as functional pathways are approached in real time. Both awake and asleep anesthesia regimens are utilized depending on the type of testing required to assess and preserve functional areas during tumor resection. Postoperatively, deficits are assessed using MRI along with clinical exam to predict whether they will be temporary or permanent.

**Conclusion:**

The standard of care for all gliomas is maximal safe resection. In this review, we describe brain mapping methods that have been developed, refined, and utilized over decades at a single institution, which have allowed us to achieve this goal safely.

## Introduction

The primary objective of glioma surgery is to achieve maximal cytoreduction with preservation of function [1–4]. However, the concept of maximal resection is constantly evolving as modern studies continue to redefine tumor boundaries and what could be safely resected. Currently, the gold standard treatment for newly-diagnosed adult-type diffuse glioma is maximal resection of contrast-enhancing disease, when present, as well as non-contrast-enhancing, FLAIR borders [5, 6]. Similarly, for *IDH* mutant astrocytomas and oligodendrogliomas, supratotal resection beyond the FLAIR margin results in lower risk of tumor recurrence, malignant transformation, and longer overall survival [7–9]. The expectation for increasingly larger tumor resections creates a greater challenge for neurosurgeons as it may increase the risk of new or worsened neurological deficits, especially in tumors within or adjacent to functional cortex. Regardless, functional status should not be compromised to achieve maximal tumor resection as postoperative neurological morbidity, especially motor deficits, are associated with worse overall survival even if maximal resection is achieved [10–12]. Gerritsen et al. recently demonstrated this by developing a new classification scheme for glioblastoma resections based on the degree of resection and functional status called the onco-functional outcome (OFO) [13, 14]. In their study, OFO1 patients had complete resection without neurological deficits and the best outcomes with a median survival of 19 months. These outcomes were significantly better than patients with OFO2 (incomplete resection without deficits) and OFO3 (complete resection with deficits), both of whom had similar survival to each other despite differences in extent of resection. Most importantly, OFO1 was most commonly achieved when intraoperative motor and/or language mapping was utilized, thereby demonstrating the importance of knowing how to correctly perform intraoperative functional brain mapping as a fundamental component of glioma surgery.

**Fig. 1:**
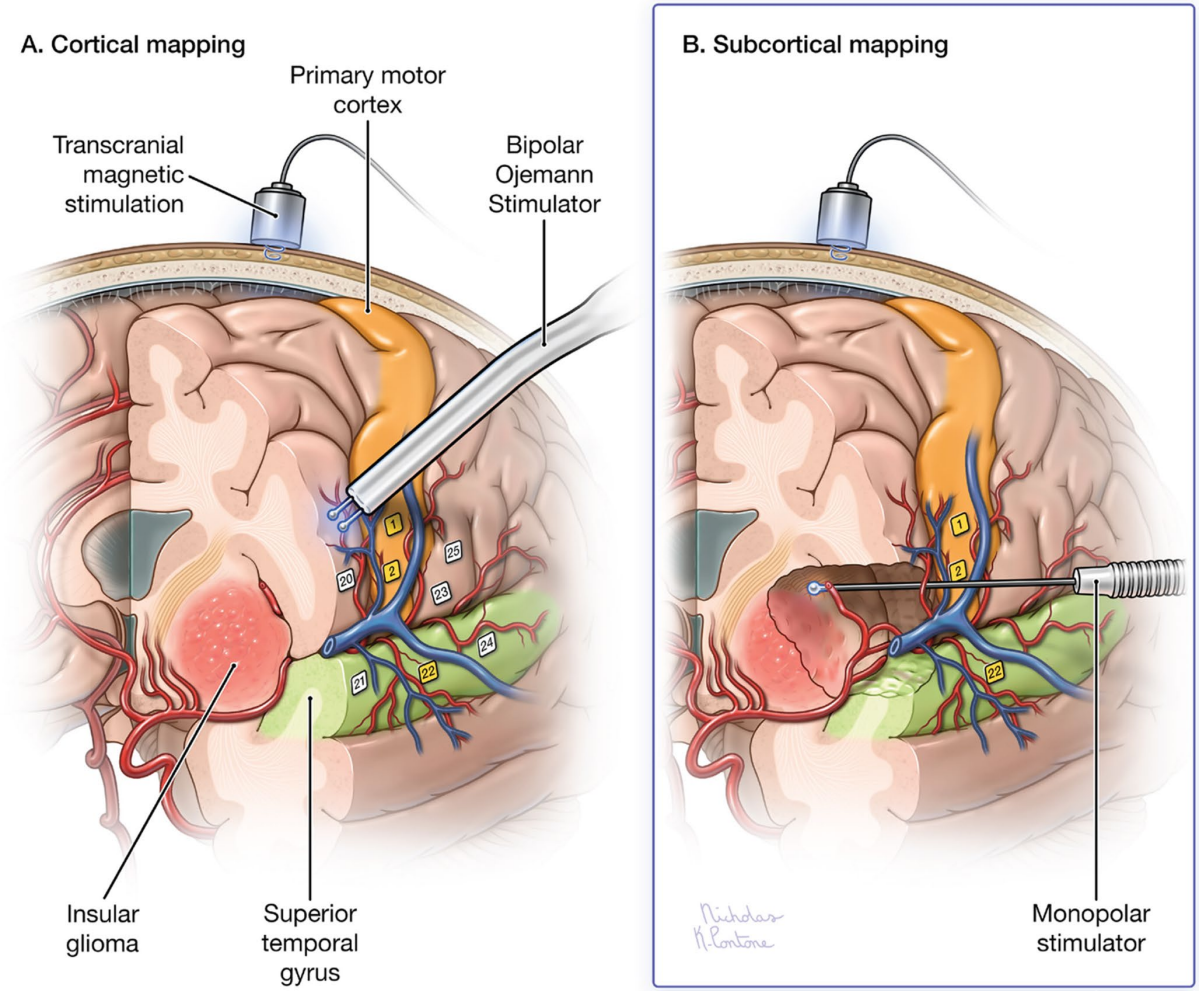
Illustration depicting the resection of a left-sided insular glioma with intraoperative language and motor mapping using (**A**) bipolar cortical stimulation to identify a negative safe-entry zone around the primary motor cortex (shaded in yellow) and superior temporal gyrus (shaded in green) and (**B**) monopolar subcortical stimulation to identify motor and language white matter tracts that should be avoided during tumor debulking

Intraoperative functional brain mapping is the accepted standard for identifying motor and language pathways and avoiding iatrogenic neurological deficits during tumor resection. In appropriately selected patients with tumors involving motor and/or language areas, intraoperative brain mapping is associated with fewer neurological deficits, higher rates of maximal resection, and shorter hospitalizations which translate into longer progression-free and overall survival, better performance status, and greater quality of life [15–17]. With increasingly more data supporting the use of intraoperative brain mapping, there has been a greater need for technical reviews describing its optimal implementation [18]. There are several reported techniques with slight variations for both language and motor mapping [19–21]. This review aims to describe the protocol for intraoperative language and motor mapping at our institution, which has developed over decades the awake “negative” language mapping paradigm and the asleep triple modality motor mapping technique, both of which limit the borders of the exposure to the boundaries of the tumor, not to the inclusion of positive mapping sites beyond the tumor, and can result in cases where no eloquent tissue is encountered at all [22–24]. For mapping motor pathways, we have transitioned our practice over time to using asleep conditions when possible, given the accuracy and safety of the asleep triple motor mapping technique. Background noise seen in the awake setting is minimized and patient comfort is improved. The risk of intraoperative seizures is also lower, and asleep conditions allow for transcranial motor evoked potentials (MEPs) to be used, which are not feasible when patients are awake. Additionally, we perform direct electrical stimulation with both low-frequency bipolar and high-frequency monopolar stimulation to maximize the advantages and offset the limitations of both modalities (Fig. 1). We also map tumors involving the supplementary motor area (SMA) under asleep conditions because in our experience and others, awake mapping of the SMA can result in premature termination of the resection without preventing an SMA syndrome, and postoperative SMA syndromes will resolve to the point where it does not significantly affect the patient [25–27]. By describing the most up-to-date protocols that are currently practiced at our institution and their rationale, the goal is to make intraoperative brain mapping more accessible and acceptable to neurosurgeons throughout the world and to feel confident in maximizing extent of resection while minimizing morbidity.

## Preoperative preparation for intraoperative mapping

For both awake language mapping and asleep motor mapping, baseline preoperative imaging at our institution consists of (1) MRI with and without gadolinium and fiducials for neuronavigation protocol; (2) diffusion tensor imaging (DTI) of functional white matter tracts; and (3) magnetoencephalography (MEG) for functional connectivity maps [28]. Acquisition of preoperative DTI and MEG is critical because there is high interindividual anatomical variability in the location of functional regions. Additionally, functional white matter tracts may be distorted by tumor mass effect, and functional cortex can shift away from conventional locations due to neuroplasticity [29–31]. Thus, MEG and DTI together are good approximations of cortical function and subcortical tract localization, which is helpful for planning the surgical approach, craniotomy boundaries, and where to apply intraoperative mapping.

**Fig. 2:**
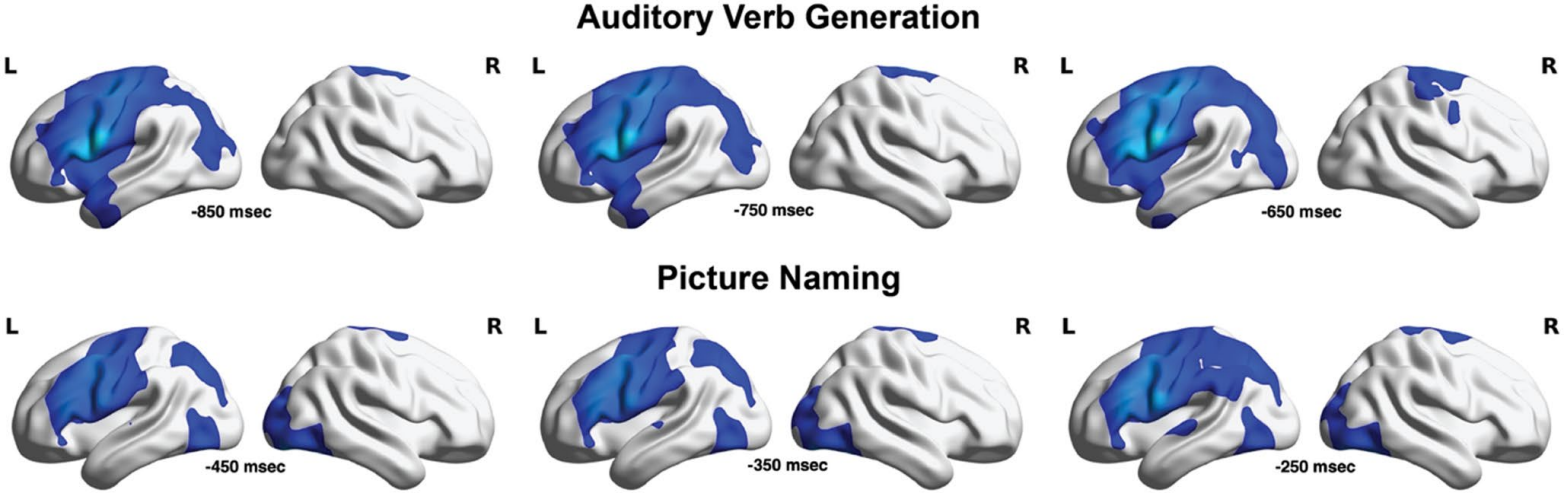
An example of magnetoencephalography results in a right-handed patient with a left-sided glioma who demonstrates preferential activation of the left inferior frontal gyrus, middle frontal gyrus, and superior temporal gyrus within 650–850 msec of stimulus during auditory verb generation and activation of the left occipitotemporal cortex, superior parietal, and premotor and inferior frontal gyrus within 250–450 msec of stimulus during picture naming, suggesting left lateralized language function

DTI and MEG are complementary methods, as DTI provides strictly anatomic information on the location of white matter tracts, whereas MEG maps real-time brain function. Figure 2 provides an example of MEG in a glioma patient with left lateralized language function, with the specific functional cortical areas precisely highlighted to help with preoperative planning. Preoperative DTI and MEG have been shown to help increase rates of gross total resection, decrease rates of postoperative deficits, improve postoperative Karnofsky Performance Status, and prolong overall survival by approximating distance from tumor to functional cortex and informing us when we need to start using intraoperative mapping, especially in cases with intra-axial masses that are nearby motor and language pathways [32–36]. Functional MRI (fMRI) is another modality for preoperatively identifying functional cortical areas [37]. However, when comparing MEG and fMRI, MEG more reliably localizes functional cortex than fMRI, with a greater specificity for identifying both motor and language areas within 20 mm of intraoperative mapping results [38–40]. This disparity is thought to exist because MEG directly measures neuronal activity to the millisecond (Fig. 2), whereas fMRI measures brain metabolism and hemodynamics (i.e. BOLD signal), which is an indirect measure of brain activity that does not reflect real-time changes in neurophysiology [39].

Although preoperative imaging with DTI, MEG, and/or fMRI improves patient outcomes and helps with surgical planning, it cannot replace intraoperative functional brain mapping, as there is a margin of error intrinsic to all preoperative imaging modalities. DTI fiber tract reconstruction can vary significantly depending on the type of algorithm and the parameters used during reconstruction, and as a result, DTI has been shown to both underestimate or overestimate the abundance of peri-tumoral white matter tracts when different methodologies are utilized [41–43]. In our protocol, we use a standardized DTI algorithm published by Berman et al. with 55–64 diffusion gradients, repetition time of 6100–8400 s, echo time of 74.5–92 s, and a B-value of 2000 [44]. For MEG, when it does identify a functional cortical area, the median offset of MEG results to functional cortex determined by intraoperative mapping has been reported to be ~ 12–19 mm [40, 45]. fMRI has also been shown to localize a median distance of ~ 5–10 mm away from intraoperative mapping, and it is less accurate than MEG, with studies demonstrating that fMRI accurately locates motor cortex in as low as 73% of patients, and language cortex in as low as 43% [39, 41, 46]. Furthermore, on top of inherent technological limitations with these modalities, intraoperative brain shift from patient positioning, operative manipulation, tumor resection, and CSF drainage can make neuronavigation inaccurate and unreliable [47, 48]. For all these reasons, it is imperative to use these preoperative imaging modalities in conjunction with intraoperative mapping. As expected, the combination of both preoperative and intraoperative mapping has been shown to increase rates of gross total resection and decrease rates of morbidity compared to use of either method alone [49].

When deciding whether to perform awake or asleep mapping based on the preoperative imaging, tumors involving the primary motor cortex and corticospinal tracts can be reliably localized under asleep conditions with an anesthesia regimen that avoids propofol and hypothermia (< 36 °C) [19, 50, 51]. Speech and language function, as well as sensory pathways, must be tested while awake and should be considered when operating in the dominant (i.e. left hemisphere in a right-handed individual) perisylvian and insular areas [22, 52–57]. Similarly, any tumor resection involving subcortical speech and language pathways should be done under awake mapping conditions [58–63].

For language mapping, preoperative language testing must be performed and includes naming objects, reading single words, and sentence generation. Objects, words, or tasks that the patient has difficulty performing preoperatively should be excluded so that intraoperative testing only utilizes tasks that the patient can complete reliably [28, 64]. If a patient being considered for awake mapping has significant mass effect or severely impaired preoperative language function (defined as > 35% naming errors), they should undergo hospitalization for a 2–3-day course of high-dose dexamethasone (4-6 mg every 6 h) and intravenous mannitol treatment (150mL of 20% every 8 h) to reduce cerebral edema. If the preoperative language testing improves to the point of making less than 35% errors and/or the motor deficit improves to anti-gravity strength, then the patient can undergo awake testing as described. If deficits do not improve with this regimen, patients should be considered for EEG monitoring to rule out language-related seizure activity. Additionally, tumor in non-functional areas can first be debulked while asleep and then if the patient recovers to a functional state as previously described, then they could undergo a second procedure under awake conditions to resect the residual tumor in functional areas [21, 28]. Patients who have persistent language deficits or hemiparesis with less than antigravity motor function, despite medical optimization and/or staged debulking while asleep, should not be considered for intraoperative mapping. In some patients with language dysfunction that does not improve, the patient could be offered awake “conversational” mapping to attempt to maintain what function does exist at baseline. It should be mentioned that intraoperative functional brain mapping is not exclusively confined to motor and language function and can be applied to higher-order functional networks including information processing speed, working memory, executive functioning, motor planning, and visuospatial cognition [65, 66]. Mapping of higher-order functional networks is not routinely performed at our institution during glioma surgery and is not part of the senior author’s standard protocol, hence why it will not be discussed in this article, however, it should be noted that it could play an important role in achieving onco-functional balance and minimizing postoperative deficits that may impact patient quality of life [64].

## Anesthesia considerations for intraoperative mapping

**Fig. 3:**
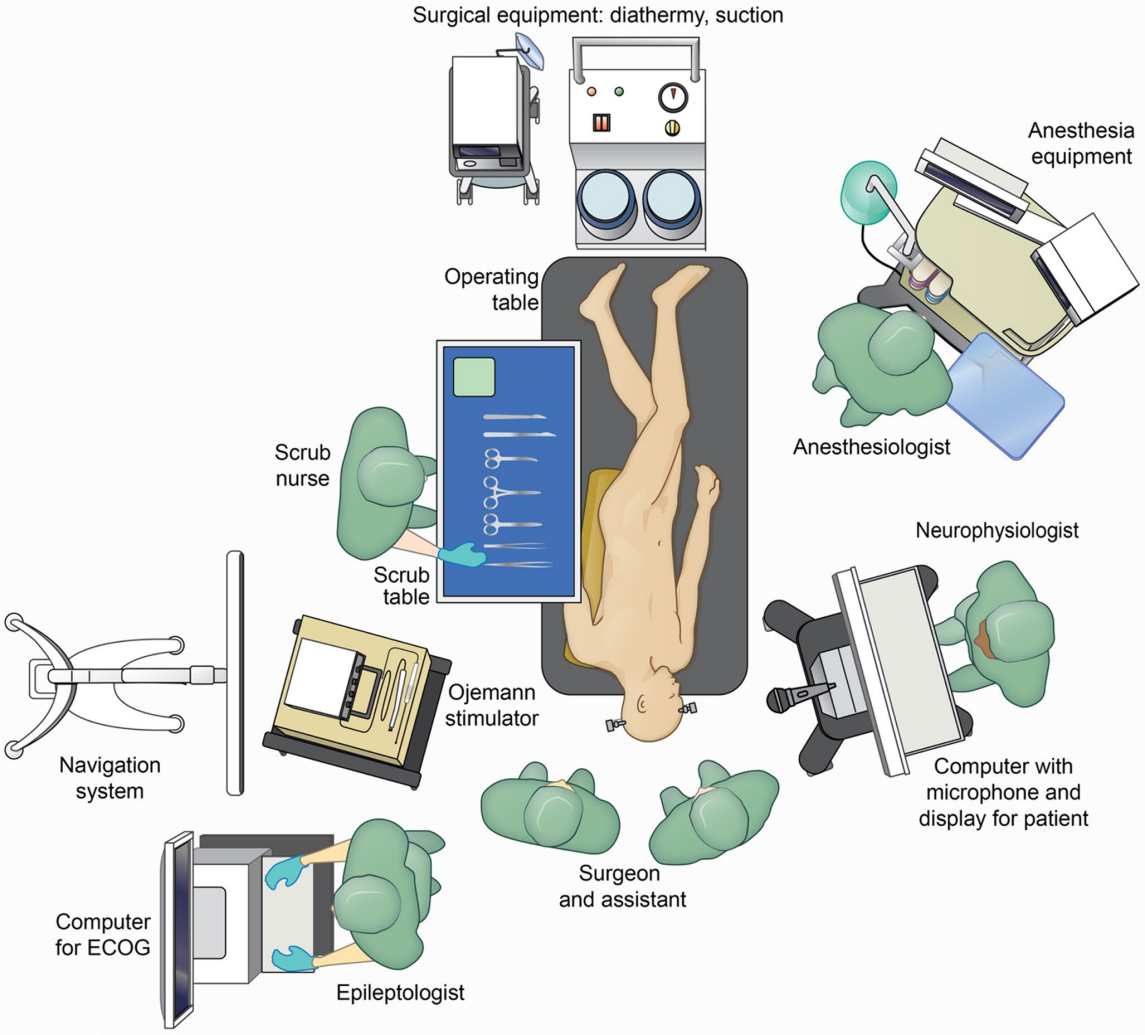
Illustration of the standard operating room layout for performing awake language mapping craniotomies at our institution

The anesthesia team plays an important role in the safe and effective implementation of intraoperative brain mapping. As shown in Fig. 3, intraoperative brain mapping is a large, coordinated effort that requires very specific personnel, equipment, and organization to maximize surgeon efficiency and patient safety. Even prior to anesthetic induction, decisions regarding access and positioning can affect the efficacy of mapping [28]. Especially in cases of motor mapping, all access lines, blood pressure cuffs, and pulse oximeters should be placed on the non-mapping, ipsilateral limbs to prevent interference and restrictions with motor evoked potential (MEP) and electromyography (EMG) monitoring [23]. The target body temperature should be greater than 36 °C in order to prevent changes in axonal depolarization that lead to unreliable motor mapping results. Patients are typically positioned in a semi-lateral position with the head turned contralateral to the side of the tumor, always facing the anesthesia team, and with minimal neck flexion in awake cases to reduce the likelihood of airway obstruction and facilitate easy placement of a laryngeal mask airway (LMA) if needed (Fig. 3). For asleep motor mapping cases, endotracheal intubation with multiple bite blocks and careful tongue position in the midline to prevent tongue laceration during mapping can be performed under general anesthesia with select agents.

**Fig. 4:**
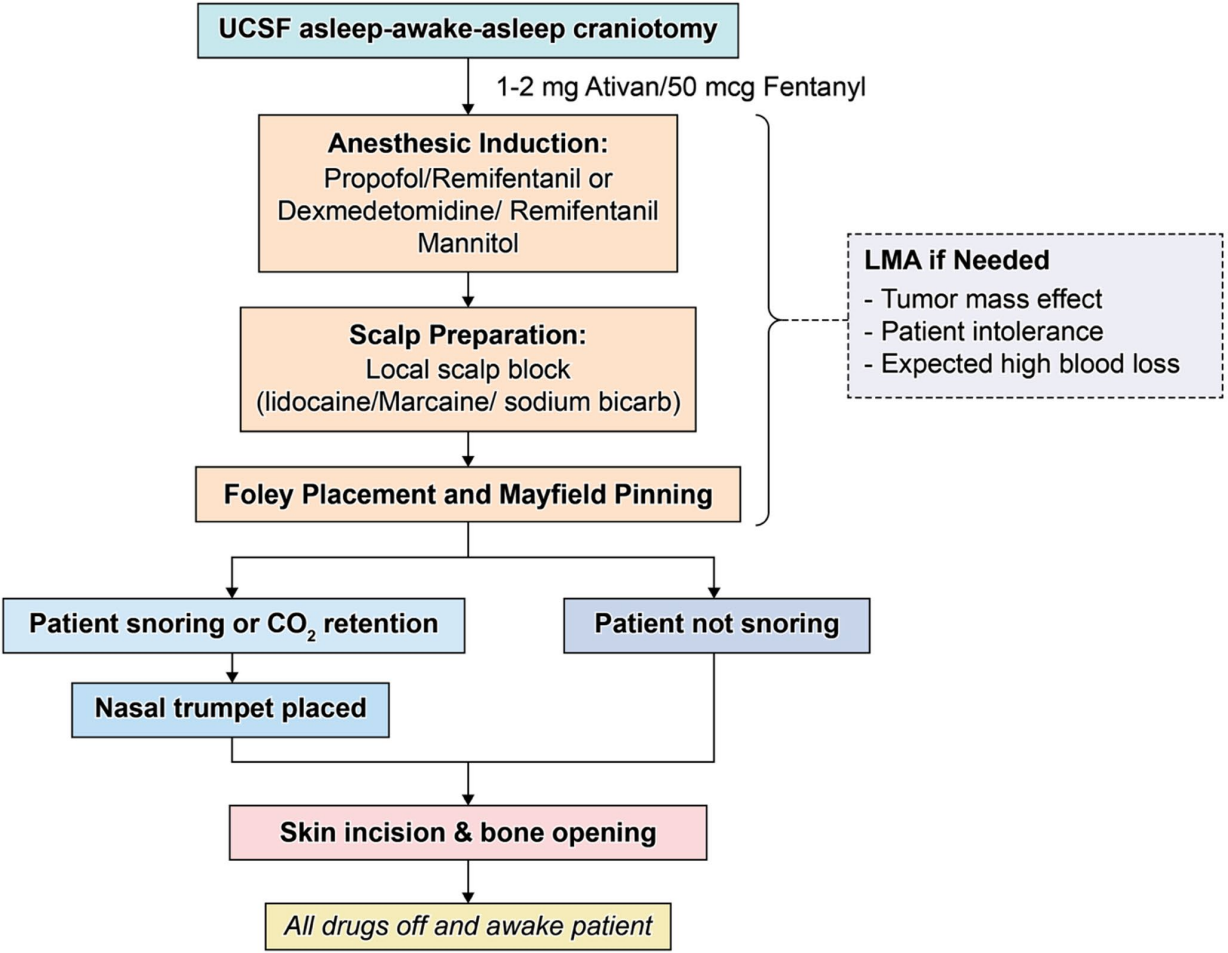
Diagram describing the standard anesthetic regimen and workflow for asleep-awake-asleep craniotomies at our institution

The selection of appropriate anesthetic agents is critical to successful intraoperative mapping. For asleep motor mapping, induction and intubation can be performed with a short-acting neuromuscular blockade and propofol sedation. However, it is essential that after induction in asleep cases, the propofol is stopped as it interferes with motor mapping due to changes in axonal depolarization. After induction, this can be followed by a maintenance anesthesia regimen throughout the case and mapping with remifentanil, 50–70% nitrous oxide, and a halogenated anesthetic such as sevoflurane, desflurane or isoflurane at a dose less than 0.5 minimum alveolar concentration [23]. There is more variety in the anesthetic regimen for awake brain mapping, which can be divided into “asleep-awake-asleep” or “awake-awake-awake” paradigms. Both techniques have their different advantages and disadvantages, and ultimately the decision is based on surgeon-preference with both protocols used equally across institutions [67]. In “asleep-awake-asleep” protocols, general anesthesia with an LMA or deep sedation without an LMA is used for the portions where the patient is asleep, which optimizes patient comfort during painful, non-mapping portions. Additionally, there is less time constraint on the surgeon during tumor resections of noneloquent areas and closing, thereby creating a more controlled atmosphere for both the surgical and anesthesia teams. However, the surgery can be delayed if there is a longer awakening time during anesthetic reversal as well as have confounded mapping results from lingering anesthetics causing extended sedation and altered consciousness [68]. In “awake-awake-awake” protocols, conscious sedation during all portions of the surgery result in fewer opioid and vasoactive medications, which translate to shorter operative times, more reliable mapping, and decreased postoperative length of stay [69, 70]. However, there is increased risk for intraoperative agitation, seizures, and hypertension, which could result in patient injury, aborted mapping, and/or intraoperative hemorrhage. At our institution, we typically use the “asleep-awake-asleep” protocol to maintain a level of sedation during the first and third portions of the case for patient comfort, and, complete wakefulness during the second portion of the case for mapping language function. A flow diagram describing the sequence of steps in our “asleep-awake-asleep” protocol leading up to awake mapping is outlined in Fig. 4. To minimize intraoperative nausea, anxiety, and pain, all patients are premedicated with 4 mg ondansetron, 2-4 mg dexamethasone, 50ug fentanyl, and a local field block with 1:1 mixture of 1% lidocaine with 1:100,000 epinephrine and 0.5% bupivacaine along the planned incision and the Mayfield pin sites. For foley placement and pinning of the head, low dose propofol is utilized to ensure patient comfort. During the non-mapping portions of the case, sedation is achieved using a target-controlled infusion of remifentanil at a rate of 0.05-0.1ug/kg/min and/or dexmedetomidine at a rate of 0.7-2.0ug/kg/min [70, 71]. Propofol can also be used at a rate of up to 100ug/kg/min depending on the patient’s tolerance to dexmedetomidine. However, it is typically avoided, if possible, given its more sedating effect, which can create respiratory issues and make mapping less reliable. During intraoperative mapping, dexmedetomidine and propofol is turned off at least 15 min prior to testing. Anxiety and pain are managed with as needed low-dose remifentanil infusions, additional local anesthetic, and/or IV paracetamol and acetaminophen. An LMA is typically used on an as-needed basis when patients are demonstrating signs of obstruction, apnea with concerns for airway protection, or significant intraoperative mass effect requiring controlled hyperventilation to reduce cerebral swelling and promote brain relaxation. Typically, a nasal trumpet can be trialed first with good effect in patients who demonstrate obstruction or snoring with sedation. LMA placement is preferred over intubation in the setting of respiratory complications given less laryngeal stimulation that could induce coughing or brain swelling and more feasible airway access given the need for fiberoptic intubation when endotracheal tubes are utilized [72].

In addition to airway complications, one of the most important complications to anticipate and promptly manage for both the surgical and anesthesia team are seizures. Intraoperative focal seizures are one of the leading causes of mapping failure with an incidence rate ranging from 3.4 to 12.6% [73, 74]. Seizures most commonly occur during cortical stimulation, hence the importance of loading, but not infusing, propofol in a large bore IV within 6 inches of the vein prior to mapping as well as having iced lactated ringer solution on standby for direct application to the cortical surface in the event of stimulation-induced focal seizures [75, 76]. If seizures occur and there is ongoing concern for subclinical seizures that are affecting intraoperative mapping, electrocorticography (ECoG) is then utilized to detect after-discharge potentials to guide further intraoperative management with anti-seizure medications and optimize stimulation thresholds so that more seizures are not encountered and false-positive mapping is avoided. Using the asleep-awake-asleep protocol and aforementioned counterstrategies, the senior author only encountered one airway complication that required conversion to an LMA and has never needed to abort a case due to intraoperative seizures refractory to immediate cold lactated ringer irrigation or medical management.

## Intraoperative motor mapping technical notes

Traditionally, intraoperative motor mapping has been most commonly performed via direct, low-frequency bipolar stimulation at both the cortical and subcortical level. This technique results in reliable identification of motor cortex but has variable identification of subcortical motor tracts, with a large series of 702 cases from our institution only resulting in mapping of the descending pathways in 43% of the cases [24]. As a result, our institution has transitioned towards a triple modality protocol for motor mapping that leverages the advantages of low-frequency bipolar stimulation, high-frequency monopolar stimulation, and transcranial or direct cortical motor evoked potential (tcMEP and dcMEP) monitoring, all of which are illustrated in Fig. 1, to enable more safe and reliable identification of motor tracts [23].

**Fig. 5:**
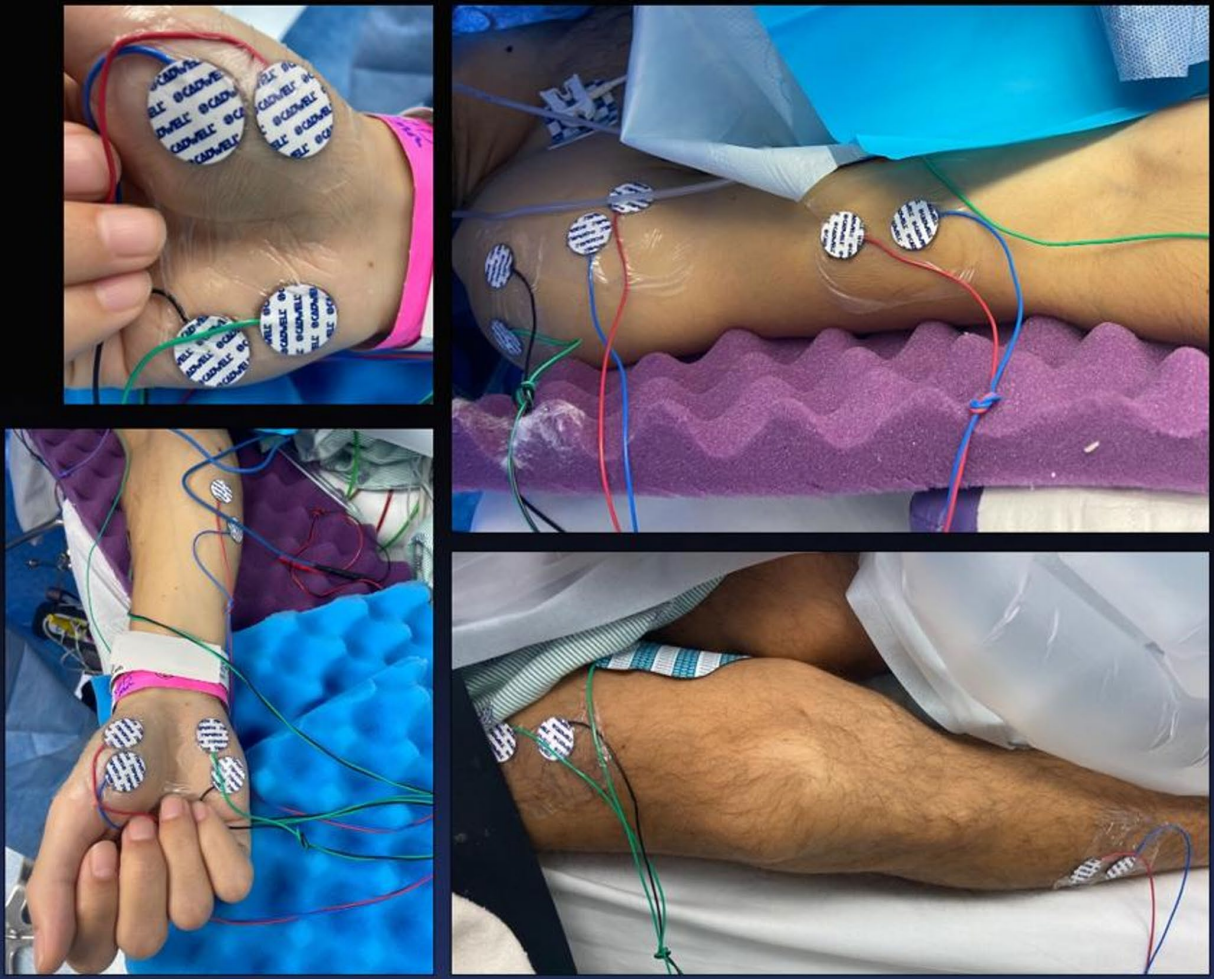
Example of adherent electromyography (EMG) pads that avoid subdermal needle puncture and can be used in awake intraoperative brain mapping cases to minimize patient discomfort

MEP monitoring is intended to provide real-time feedback on the integrity of the corticospinal tract throughout brain mapping and tumor resection. tcMEPs are motor responses generated from a corkscrew electrode on the scalp that passes a stimulus through the skull to the motor cortex (Fig. 1). This allows for testing of both cortical hemispheres and compares the motor cortex being operated on to the intact contralateral hemisphere, which is a more specific measurement if there has been damage to the motor pathways. dcMEPs are motor responses generated from a cortical strip electrode on the motor cortex, which cannot always be placed depending on the craniotomy, but can allow for continuous stimulation if utilized and will be a more sensitive and immediate signal if the motor tracts are disrupted and the signal drops [48]. The tcMEP corkscrew is placed immediately after neuronavigation registration is complete and the scalp site directly overlying the motor cortex can be identified (Fig. 1). Subdermal EMG needles must also be placed preoperatively in at least the contralateral face, upper arm, forearm, hand, upper leg, lower leg, and foot to enable monitoring. Of note, in cases with awake motor mapping, sticky EMG pads are used in lieu of needles to minimize patient discomfort. An example of these adherent EMG pads is shown in Fig. 5. MEP monitoring is typically performed with a train-of-4 or 5 pulse lasting 0.5ms and an interstimulus interval of 4ms. The intensity of the pulse is titrated to the lowest effective intensity required to elicit a motor response in order to increase the sensitivity of motor tract damage and avoid false negative signals [77]. In asleep cases, the intensity for tcMEP and dcMEP can be as high as 200 mA and 20 mA, respectively. The interpretation of tcMEP and dcMEP responses are crucial for safe intraoperative motor mapping. Typically, a greater than 20% increase in stimulus intensity threshold or a greater than 50% irreversible loss in MEP amplitude is a strong indicator of injury to the motor pathways and associated with permanent postoperative paresis [77]. In these situations, the resection and mapping should be paused and a systematic evaluation of possible reversible confounders including changes in anesthesia regimen, patient temperature, blood pressure, or brain sag secondary to tumor resection causing suboptimal corkscrew or strip electrode placement should be performed.

Once MEP monitoring is established, direct cortical stimulation for the actual motor mapping can then take place. The goal of direct stimulation is to identify the lowest electrical intensity required to produce an MEP at a cortical or subcortical location suspected to be a part of the motor pathway. A site is positive if the lowest electrical intensity is below a certain threshold and negative if no response is elicited above a certain intensity. By defining positive and negative mapping sites, the boundary of the motor cortex and tracts can be delineated and guide where it is safe to resect. Low-frequency bipolar stimulation with a 5 mm probe (i.e. 5 mm space between bipolar probe tips), as shown in Fig. 1A, delivering a 60 Hz pulse for 1ms starting at an intensity of 2-4 mA and up to a maximum of 16-18 mA in asleep patients (3-4 mA in awake patients) is the standard technique for cortical mapping and determining the negative safe-entry zone. Typically, a cortical site is positive if a motor response is elicited with an intensity ranging between 2 and 6 mA, although this threshold is higher when lesions are in subcortical descending motor fibers [20]. The general stimulation parameters for our institution’s mapping protocol are described in Table 1. Low-frequency bipolar stimulation is generally preferred for cortical mapping because the charge is more localized and contained within the two probe arms, whereas high-frequency monopolar stimulation has a more diffuse electrical field and thus is more likely to identify false positive sites [78]. However, it should be noted that there is growing evidence to suggest that high-frequency monopolar stimulation may be indicated for cortical mapping in select cases, specifically at the beginning when deciding which region to hone in on with the bipolar stimulator or in tumors that are directly in the primary motor cortex. When the tumor is directly within the primary motor cortex, there is a much higher risk of false negative mapping and intraoperative seizures because higher currents and longer stimulation durations are required to elicit a positive response [79]. Alternatively, a standard high-frequency monopolar train-of-5 stimulus has been shown to more reliably identify the negative safe-entry zone in M1 tumors. Furthermore, the high-frequency stimulus can be modulated depending on the characteristics of the tumor. An increased train-of-7 stimulus is able to reliably map recurrent tumors with significant scarring from prior resections and radiation treatments, while a decreased train-of-2 stimulus is able to map lower grade tumors with highly irregular borders and no contrast-enhancement more reliably [80, 81]. Overall, high-frequency stimulation for cortical mapping is gaining popularity and utility in this modern era.

**Table 1:** Stimulation conditions for asleep triple motor and awake language mapping

	Asleep Triple Motor Mapping				Awake Language Mapping
	Cortical and Subcortical		MEPs		Cortical and Subcortical
Probe	Low frequency bipolar probe	High frequency monopolar probe	High frequency corkscrew electrode (tcMEP)	High frequency strip electrode (dcMEP)	Low frequency bipolar probe
Pulse Polarity	Biphasic	Anodal - Cortical Cathodal - Subcortical	Anodal	Anodal	Biphasic
Pulse Duration Range	500–1000 usec	500–800 usec	50–500 usec	50–500 usec	500–1000 usec
Stimulation Frequency Range	60 Hz	250–500 Hz	200–1000 Hz	250–500 Hz	60 Hz
Stimulation Intensity Range	6–16 mA	2–20 mA	0–200 V	2–20 mA	2–4 mA

Finally, once the cortical window is completed over the safe cortical entry site and the surgeon is ready to begin subcortical tumor resection, motor mapping transitions entirely to high-frequency monopolar stimulation at our institution, as shown in Fig. 1B [23]. This is because the more diffuse nature of monopolar stimulation allows for motor fiber detection up to several millimeters away from the resection and stimulation site, thereby enabling more complete characterization of all the surrounding motor fibers and a safer resection [23]. As previously mentioned, the standard monopolar stimulation technique is with a monophasic train-of-5 pulse up to 500 Hz for a duration of 500us starting at an intensity of 10-20 mA and decreasing incrementally to as low as 1-3 mA to identify the minimal motor threshold [20, 82, 83]. The relationship between stimulation intensity and distance is linear whereby a threshold of 1 mA is approximately equivalent to 1 mm distance from the motor tracts. Thus, a site is generally considered positive and should signal to the surgeon to start limiting the resection if a response is elicited with a current between 2 and 5 mA, which signals a distance of 2–5 mm from the nearest subcortical motor tract. It is crucial to constantly alternate between tumor resection and subcortical stimulation in order to avoid inadvertent injury to the surrounding subcortical motor pathways. Given the threshold range of 2-5 mA, a good rule of thumb is to reperform subcortical stimulation after every 2–3 mm of resection. This can be attenuated with a train-of-2 pulse sequence to allow for an even closer determination of the motor tract.

## Intraoperative language mapping technical notes

If mapping both motor and language function in the same awake operation, it is recommended to perform motor mapping first because movement is easier to test and observe as the patient awakens from anesthesia [84]. When the patient is alert and ready for language mapping, cortical testing sites suspicious for language and speech function are identified and should be separated by 1 cm. Each site should be tested at least three times with alternation of sites such that the same area is never stimulated twice successively [22, 28, 48]. The traditional technique for intraoperative language mapping is via low-frequency bipolar stimulation with 1.25ms biphasic square waves delivered in 4-second trains at 60 Hz [28]. Recent data has emerged to suggest that high-frequency monopolar stimulation with a 250–500 Hz monophasic train-of-5 pulse is non-inferior to bipolar stimulation for language mapping [85, 86]; however, bipolar stimulation remains the more commonly used modality [21]. With the low-frequency bipolar technique, mapping of the cortical surface should begin with a stimulation intensity of 2 mA for 3–4 s while the patient is completing a language mapping task. Tasks should be separated by 4–10 s, and the stimulation intensity should be increased until either a positive site is identified, an after-discharge is encountered, or a maximum stimulation intensity of 4 mA is reached [28, 48]. Common tasks include picture naming, counting, word repetition, reading, and language syntax, and should be tailored to the stimulated region [21]. A positive map is a site with a > 65% error rate, defined as either speech arrest (without simultaneous motor deficits), anomia, alexia, or semantic or phonological paraphasia on at least two of three trials [22, 87, 88]. Positive sites should be marked with numbered tags. If an after-discharge is encountered, the stimulation intensity should be decreased by 1 mA to avoid after-discharge-induced errors and prevent seizures [48]. As previously mentioned, ECoG monitoring with an epileptologist during awake stimulation mapping can help identify persistent subclinical seizure discharges that can cause false positive events on testing. The presence of a trained neuropsychologist/neurolinguist is essential to safely and effectively performing language mapping, preferably the same individual who performed the preoperative testing.

After mapping the exposed cortical surface, cortical dissection and resection of negative, function-free sites can take place. A 5–10 mm margin of tissue should be preserved around all positive cortical sites to protect language function [28]. After cortical resection, language mapping of subcortical structures can utilize the same stimulation technique and parameters as cortical mapping, which is in contrast to motor mapping where cortical and subcortical mapping require different parameters as previously described [89]. With subcortical resection, similar to motor mapping, it is important to alternate between stimulation/testing and tumor resection in order to prevent iatrogenic injury, and in our institutional protocol, we will preserve a 2 mm margin from positive subcortical structures using the 1 mA per 1 mm train-of-5 protocol. In patients who experience intraoperative and long-term deficits, the majority are encountered during subcortical dissection with positive mapping. Those events may be associated with postoperative DTI changes in the subcortical fibers, suggesting that manipulation of the tracts itself can cause direct damage, hence the importance of meticulous and intermittent stimulation and resection while working in these subcortical areas, along with avoidance of coagulation [90]. However, it is also important to be thoughtful and balanced about where mapping is applied and limit its use to regions with a high pre-test probability of function as prolonged and/or unnecessary mapping can increase patient fatigue and discomfort.

Throughout intraoperative language mapping, it is imperative to use one’s understanding of functional anatomy in combination with intraoperative neuronavigation to guide the language tasks being performed and the interpretation of errors. Naming errors can be broadly divided into semantic paraphasias (substitution of intended word with a related word) and phonological paraphasias (substitution, omission, or addition of a sound/phoneme to intended word). The former is associated with ventral language pathways below the superior temporal sulcus and the frontal lobe including the inferior frontal gyrus, anterior superior and middle temporal gyrus, inferior fronto-occipital fascicle (IFOF), and uncinate fasciculus. The latter is associated with dorsal streams including the supramarginal gyrus, arcuate fasciculus (AF), and superior longitudinal fasciculus (SLF). Repetition errors including conduction aphasia are associated with the posterior superior temporal and supramarginal gyrus and the AF and IFOF. Reading errors including alexia are associated with the superior temporal gyrus, supramarginal gyrus, and midfusiform gyrus, as well as the AF and inferior longitudinal fasciculus (ILF) [21]. Specific language errors incongruent with the area being tested warrant re-evaluation to either confirm if it is a positive map, if the current localization is accurate, or if there are subclinical seizures with ECoG.

Postoperatively, for both motor and language mapping, the standard protocol is to acquire an MRI with and without contrast and with DTI within 48 h of surgery to not only evaluate for extent of resection but also DWI signal to identify ischemic injury or DTI tract discontinuity that could explain any new neurological deficits [22]. Having both DWI and DTI postoperatively is important for understanding intraoperative findings and how to prognosticate long-term recovery, as significant DWI restriction in subcortical white matter adjacent to the resection cavity or DTI discontinuities can be supportive of both immediate and long-term neurologic deficits [90]. However, it should also be noted that there is growing evidence that the severity of diffusion imaging abnormalities does not directly correlate to the severity and/or potential for recovery of postoperative deficits, as surgical manipulation and transient ischemia alone can lead to DWI signal, and this can be elucidated clinically with comparison to the corresponding ADC values, with lower values being less likely to be permanent ischemic changes [91].

## Conclusion

Maximal safe surgical resection is the standard of care for glioma surgery, as it has been shown to prolong overall survival and decrease risk of tumor recurrence. Function should not be compromised in pursuit of larger tumor debulking because postoperative neurologic deficits, especially motor deficits, are associated with worse survival and quality of life. Striking balance is the challenge every neurosurgeon faces when tumors invade into or around functional cortex. Intraoperative functional brain mapping remains the most effective tool for achieving maximal safe resection; however, the field is constantly evolving, and there are variations in the techniques reported in the existing literature. By detailing the most up-to-date protocols practiced at our institution, we hope that this review will serve as an effective template and guide that allows neurosurgeons everywhere to safely and successfully perform intraoperative motor and language mapping for glioma surgery.

## Data Availability

No datasets were generated or analysed during the current study.
